# High-Density Arrayed Spectrometer with Microlens Array Grating for Multi-Channel Parallel Spectral Analysis

**DOI:** 10.3390/s25154833

**Published:** 2025-08-06

**Authors:** Fangyuan Zhao, Zhigang Feng, Shuonan Shan

**Affiliations:** Tsinghua Shenzhen International Graduate School, Tsinghua University, Shenzhen 518055, China; fy-zhao24@mails.tsinghua.edu.cn (F.Z.); fzg24@mails.tsinghua.edu.cn (Z.F.)

**Keywords:** spectrometer, spectral detection, multi-channel spectrometer, MLAG, Micro-LED, line-scan spectral confocal

## Abstract

To enable multi-channel parallel spectral analysis in array-based devices such as micro-light-emitting diodes (Micro-LEDs) and line-scan spectral confocal systems, the development of compact array spectrometers has become increasingly important. In this work, a novel spectrometer architecture based on a microlens array grating (MLAG) is proposed, which addresses the major limitations of conventional spectrometers, including limited parallel detection capability, bulky structures, and insufficient spatial resolution. By integrating dispersion and focusing within a monolithic device, the system enables simultaneous acquisition across more than 2000 parallel channels within a 10 mm × 10 mm unit consisting of an *f* = 4 mm microlens and a 600 lines/mm blazed grating. Optimized microlens and aperture alignment allows for flexible control of the divergence angle of the incident light, and the system theoretically achieves nanometer-scale spectral resolution across a 380–780 nm wavelength range, with inter-channel measurement deviation below 1.25%. Experimental results demonstrate that this spectrometer system can theoretically support up to 2070 independently addressable subunits. At a wavelength of 638 nm, the coefficient of variation (CV) of spot spacing among array elements is as low as 1.11%, indicating high uniformity. The spectral repeatability precision is better than 1.0 nm, and after image enhancement, the standard deviation of the diffracted light shift is reduced to just 0.26 nm. The practical spectral resolution achieved is as fine as 3.0 nm. This platform supports wafer-level spectral screening of high-density Micro-LEDs, offering a practical hardware solution for high-precision industrial inline sorting, such as Micro-LED defect inspection.

## 1. Introduction

As a core instrument for characterizing the wavelength–intensity relationship, the spectrometer plays a crucial role in a wide range of applications, including spectral analysis of light-emitting devices, confocal microscopy, multi-component atmospheric pollutant monitoring, ophthalmic diagnostics, and climate observation [1,2,3,4,5]. Taking spectral confocal technology as an example, this high-precision, non-contact optical measurement technique leverages the wavelength-dependent focal response of a broadband light source along the axial direction [6,7,8,9]. By employing spectroscopic analysis, it decodes displacement [10,11,12,13,14,15,16,17,18], film thickness [19,20,21,22,23,24], roundness [25,26,27], and surface reflectivity [28,29,30] information that is encoded in the wavelength of the reflected light. This approach demonstrates excellent resolution and accuracy, even though many traditional and emerging techniques are also available for obtaining such measurement information [31,32,33].

To meet the demands of these diverse applications, spectrometer technology has evolved toward broader spectral coverage, miniaturization, and higher levels of integration in recent years [34,35,36,37,38]. On the one hand, advances in the fabrication of concave gratings with large groove constants and high curvature have enabled the development of compact spectrometers with broad spectral coverage [39,40,41,42,43]. Although approaches based on metasurfaces, integrated photonics, and compressive sensing have enabled compact and broadband spectrometer designs, these methods often face challenges such as complex fabrication, limited throughput, or the need for intensive computational reconstruction, which may impede their use in high-density parallel detection [3,5]. On the other hand, the rapid development of integrated photonics and multi-emitter sources—such as vertical-cavity surface-emitting laser (VCSEL) arrays, Micro-LED arrays, and spectral confocal line-scan systems—has led to a significant increase in the demand for multi-channel parallel processing aimed at planar scanning and even three-dimensional information acquisition. These trends impose higher requirements on both the measurement efficiency and data processing capabilities of modern spectrometers [44,45,46,47]. In response, researchers have proposed new array-based spectrometer architectures that enable high-throughput spectral analysis through multi-channel parallel processing [48,49,50,51,52,53].

Commercial multi-channel spectrometers typically adopt stacked architectures composed of multiple single-channel modules. Representative examples include the Ocean Optics eight-channel spectrometer (180–1100 nm, 0.1 nm resolution, 7 kg) and the HORIBA 96-channel spectrometer (360–780 nm, <3.5 nm resolution) [54,55]. These conventional designs are constrained by their bulky size, heavy weight, and limited channel scalability, making them ill-suited for high-density and highly integrated applications in industrial manufacturing. To overcome these limitations, recent efforts have focused on integrating dispersive and focusing functions within miniaturized optical components [56,57,58,59]. For example, Traut et al. [60] employed photolithographic holography to fabricate microlens–grating composite structures (resolution > 10 nm); Hirano et al. [61] constructed a blazed grating–microlens array system via physical vapor deposition and electron beam lithography; and Shi et al. [62] applied thermal reflow self-assembly to realize microlens array gratings with a resolution of 6.9 nm. However, these approaches often encounter significant technical challenges, such as the need for cleanroom conditions and material shrinkage during polymer curing. In parallel, miniature spectrometers using Fresnel lenses have been developed to further reduce system footprint and enhance compactness [63,64,65]. While such designs can achieve high spectral resolution (down to 0.2 nm), they face scalability issues when increasing the number of parallel detection channels.

Several recent strategies have been proposed to enable multi-channel parallel spectral analysis. For instance, Shan et al. [66] developed a microlens array–grating (MLAG) technique using soft lithography to integrate multiple spectral units, achieving a 220 μm channel pitch and sub-pixel spot center repeatability, thus demonstrating strong potential for high-density parallel detection. However, the system still relies on external optics to adjust divergence angles. Fathy et al. [67] proposed a multi-interferometer approach based on micro-electro-mechanical systems (MEMS) that improves spectral resolution through multipath interference, achieving up to a threefold enhancement, though its performance is limited by the thermal stability of MEMS actuators. Hybrid micro-optical systems have also been explored to bridge performance gaps. For example, Hao et al. [68] introduced a multi-aperture snapshot imaging spectrometer employing tilted linear variable filters (LVFs) and a microlens array, realizing an 11 × 6 spectral channel matrix, albeit with spectral distortion at large apertures. Similarly, Chen et al. [69] proposed an infrared array spectrometer achieving high resolution at the expense of optical throughput (i.e., photon efficiency). Collectively, these approaches underscore an enduring trade-off in multiplexed spectral sensing: scalability versus performance.

To address this challenge, we propose a novel spectrometer architecture specifically designed for multi-channel parallel spectral detection. The core element of the system is a double-sided integrated microlens array grating (MLAG) that simultaneously performs dispersion and focusing. This is complemented by a dual-microlens aperture assembly that enables flexible adjustment of the divergence angle of the incident light. A CMOS sensor serves as the detection module, while spectral data are processed by a higher-level computing system to enable multispectral co-analysis. Building upon previous work [66], this study further advances the overall optical design, experimentally validates the performance of the array-based spectrometer, and proposes an image enhancement algorithm to improve resolution. The results show that the fabricated device comprises up to 2070 spectral units, exhibiting high uniformity and consistent dispersion characteristics. Single-channel spectral measurements confirm effective dispersion, with spectral resolution at the nanometer level and repeatability better than 1 nm.

## 2. Structure and Operating Principle of Multi-Channel Spectrometers

### 2.1. Structural Composition of Multi-Channel Spectrometers

**Figure 1:**
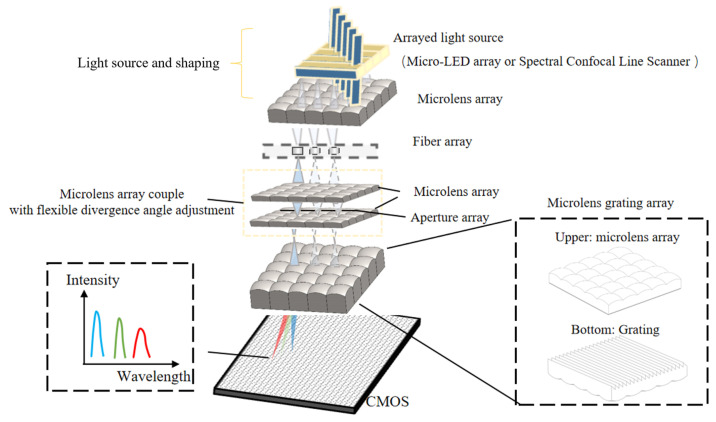
Schematic diagram of the structure of the array multi-channel spectrometer system.

Figure 1 illustrates the structural schematic of the proposed multi-array, multi-channel spectrometer system. The system is designed for testing array-based light sources, such as Micro-LED arrays and spectral confocal line-scan probes. The light source is initially focused and its sub-source size reduced through a microlens array, allowing for efficient coupling into the corresponding channels of an optical fiber array to form an arrayed light source signal. This arrayed light then passes through a scattering angle adjustment module—comprising a microlens array, an aperture, and another microlens array—which flexibly adjusts the output angles in accordance with the diameter of the MLAG subunits. As a result, an arrayed light source with a defined scattering angle is produced. Each sub-source subsequently enters its corresponding MLAG unit, where it undergoes further focusing and dispersion. The resulting spectrally dispersed light is imaged onto a CMOS sensor, enabling the acquisition of spectral signals. The spectral data from all sub-sources are collected simultaneously, thereby achieving multi-channel parallel spectral analysis.

### 2.2. Operating Principle of Microlens Array Grating Spectrometers

The fundamental working principle of a dispersive spectrometer lies in spectral dispersion—that is, separating incident light into its constituent wavelengths for analytical purposes. Such systems typically comprise three core components: an entrance slit, a dispersive element, and a detector. The entrance slit spatially confines the incoming light, ensuring well-defined beam boundaries prior to interaction with the dispersive element.

At the heart of multi-channel spectrometer functionality is the MLAG. Figure 1 illustrates the structural composition of an MLAG subunit. Each subunit consists of a microlens for focusing incident light and a diffraction grating for dispersing the focused beam into its spectral components. Due to the small aperture of the microlenses, only a limited number of grating lines are illuminated, which in turn affects the spectrometer’s resolution. To address this, a blazed grating with a blaze angle of 8°37′ and a blaze wavelength of 500 nm was employed in the experiment to enhance the intensity of the diffracted light on one side. Additionally, image processing techniques were applied to further improve the resolution.

During operation, incident light enters the MLAG system and first interacts with the microlens array. The microlenses focus the light into discrete spots, ensuring that each optical path is well-defined and spatially confined. These focused beams then pass through a diffraction grating with period *d*, where they are diffracted at angles that depend on their wavelengths. This diffraction process spatially separates the light into its spectral components, with each wavelength deflected at a characteristic angle as defined by Equation (1).

(1) dsinα±dsinθ=mλ,m=0,±1,±2…

**Figure 2:**
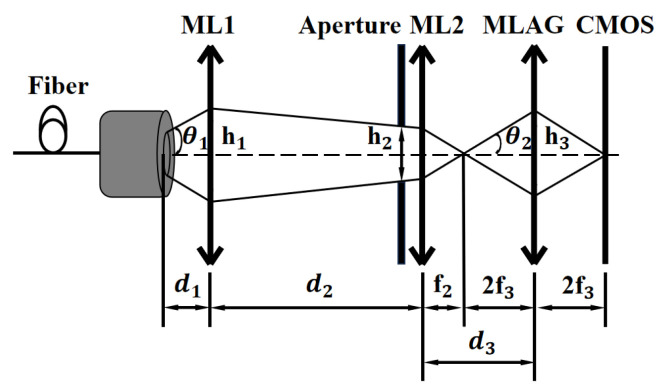
Optical path design of single channel spectrometer. $\theta_{1}$ and $\theta_{2}$ represent the divergence angles of the light beam at ML1 and MLAG, respectively; $h_{1}$, $h_{2}$, and $h_{3}$ denote the beam widths at ML1, ML2, and MLAG, respectively; $f_{2}$ and $f_{3}$ are the focal lengths of ML2 and the MLAG lens elements, respectively; and $d_{1}$, $d_{2}$, and $d_{3}$ are the distances between key components.

In addition, another key component of the array-based spectrometer is the incident light divergence angle adjustment assembly , which consists of a pair of microlens arrays and a pinhole aperture. Figure 2 illustrates the optical path for implementing a single-channel spectrometer. The laser is emitted through an optical fiber port, with an output beam diameter $h_{1}=105$ μm.

The primary function of the two microlens arrays is to adjust the divergence angle of light incident on MLAG subunits, thereby ensuring compatibility with MLAG sub-arrays of arbitrary sizes. Specifically, ML1 serves to collimate the light, which emerges from the optical fiber with a certain divergence angle, while ML2 focuses the collimated beam. By adjusting the distances between the light source and the two microlens arrays, the divergence angle of light incident on each MLAG subunit can be finely tuned. To achieve optimal image quality and prevent crosstalk between neighboring MLAG focal spots, the incident divergence angle prior to entering the MLAG should satisfy the following condition:

(2) $$\theta_{2}\leq \arctan\left(\frac{h_{3}}{2f_{3}}\right)$$

Here, $h_{3}$ denotes the width of the MLAG subunit, and $f_{2}$ represents the focal length of the MLAG lens element.

Similarly, the pinhole aperture serves to adjust the size of the optical source image to match the lens aperture of the MLAG subunits. If the focal spot is too large, it reduces the number of available channels, degrades spectral resolution, and increases the likelihood of channel crosstalk. Conversely, if the spot is too small, the light utilization efficiency decreases, and measurement repeatability is compromised.

It is important to note that the parameter values used in this study represent only one feasible configuration. In practice, these parameters can be flexibly modified and recombined as needed. The proposed divergence-angle adjustment module can be readily adapted to MLAG elements with varying sub-lens diameters, thus enabling a configurable multi-channel array spectrometer compatible with a wide range of array-type light sources.

## 3. Experimental Validation of the Array-Based Spectrometer

### 3.1. Construction and Calibration of the Array Light Source System

**Figure 3:**
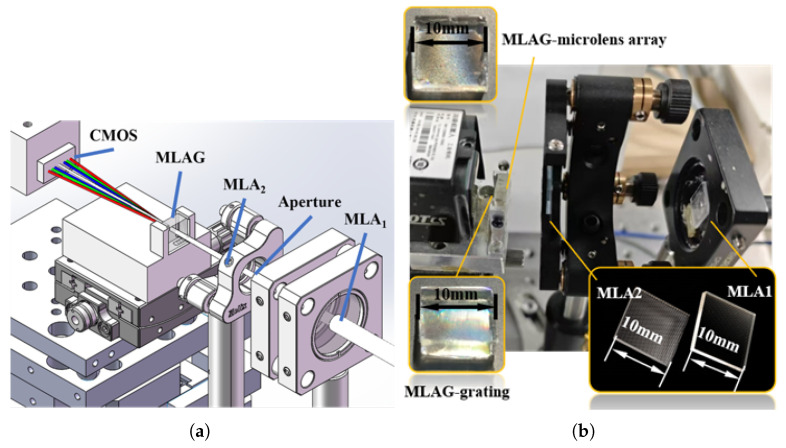
Optical path of array multi-channel spectrometer system: (**a**) optical path model of the array spectrometer system; (**b**) actual picture of the array spectrometer system. MLA: Microlens Array; MLAG: Microlens Array Grating; CMOS: Complementary Metal-Oxide-Semiconductor.

As illustrated in Figure 3a, the spectrometer system consists of a laser source, a divergence-angle adjustment module (comprising a microlens–aperture–microlens assembly), a spectral dispersion module based on the MLAG, and a CMOS detector for signal acquisition. In the experiment, the laser provides light at different wavelengths to evaluate the functional performance and uniformity of the array. The laser is coupled into the system via an optical fiber with a core diameter of 105 μm and a numerical aperture (NA) of 0.22. The light then passes through the MLA_1_–aperture–MLA_2_ module and subsequently illuminates the MLAG. The CMOS sensor captures the resulting far-field pattern, enabling analysis of focusing behavior and spot uniformity.

After assembling the microlens grating array spectrometer, it is necessary to calibrate the correspondence between CMOS pixel indices and the associated wavelengths. This is achieved by fitting a calibration curve based on measured data. In general, increasing the number of data points or using higher-order polynomials improves the fitting accuracy. However, when the curve exhibits only slight variation, higher-order fitting may offer negligible improvement in detection accuracy while incurring greater computational cost. Therefore, a trade-off must be made between accuracy and computational efficiency. In this study, three wavelengths are selected for calibration, and a second-order polynomial fitting is applied, as expressed in Equation (3):

(3) $$L=a_{0}p^{2}+a_{1}p+a_{2}$$

Here, *L* denotes the actual wavelength, and *p* represents the pixel displacement between the first-order and zeroth-order diffraction spots (i.e., the center-to-center distance between the spots on the CMOS sensor). By differentiating Equation (3), the spectral resolution corresponding to the width of a single pixel can be obtained:

(4) $$L^{\prime }=2a_{0}p+a_{1}$$

As shown in Equation (4), the focal spot position on the CMOS sensor is directly related to the wavelength of the incident light and is independent of other spatial variables. As a result, the spectral width associated with each pixel is non-uniform across the sensor.

### 3.2. Subunit Uniformity Test

**Figure 4:**
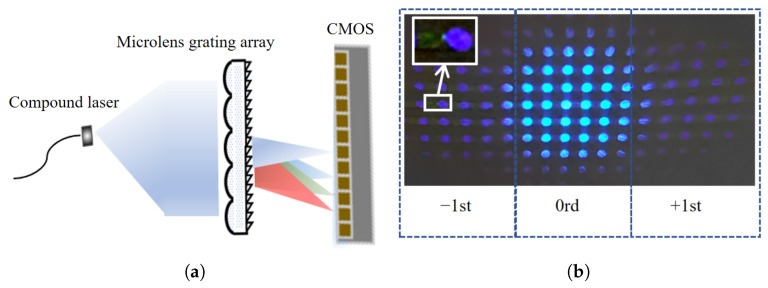
Component consistency test: (**a**) MLAG consistency test optical path; (**b**) MLAG consistency imaging results.

Since the core component of the array-based spectrometer is the MLAG, its fundamental functionality and uniformity were first verified to evaluate the consistency across individual spectrometer subunits. As shown in Figure 4a, a composite blue-green laser source was coupled into the system via an optical fiber. After collimation by a lens, the parallel beam was directed onto the MLAG surface. The resulting imaging, shown in Figure 4b, demonstrates that the parallel beam is focused on multiple sub-spots distributed across different channels with no visible crosstalk and relatively uniform brightness in the central region. On either side of the central focus, distinct ±1st-order diffraction spots are observed, indicating effective wavelength separation between the blue and green components. These results confirm the MLAG’s strong focusing and dispersive capabilities, validating its role as the core element of the spectrometer.

To quantitatively assess subunit uniformity, a single-point laser source was expanded into a regular grid of equally spaced laser spots by the MLAG, forming a uniform array pattern. The centroid position of each spot was calculated to determine pixel locations, and the distances between adjacent centroids were measured. By translating the MLAG along the x- and y-axes, the imaging uniformity of the entire device was assessed. Table 1 presents the distances between 0th-order focus and its neighboring spots in the x-direction (right) and y-direction (down) for randomly selected subunits at different positions. These values were used to evaluate the spatial uniformity of the microlens array. The coefficient of variation (CV), defined as the standard deviation divided by the mean, was used as a measure of uniformity—lower CV values indicating better consistency. The measured CV values were 1.25% in the x-direction and 1.11% in the y-direction, suggesting slightly better uniformity along the y-axis.

### 3.3. Spectrometer Channel Performance Testing

#### 3.3.1. Single-Unit Testing in the Array-Based Spectrometer

To clearly illustrate the system’s performance, we first present the spectral results of a single spectrometer subunit, which can be generalized to the array level by replacing individual components with full arrays. The test system, illustrated in Figure 3b, employs a three-wavelength composite laser source (660 nm/520 nm/450 nm). The laser is delivered through an optical fiber with a 105 μm core diameter. The first microlens array (MLA_1_) consists of subunits with a side length of 125 μm and a focal length of 0.4 mm. Given physical constraints on the fixed components, MLA_1_ serves to collimate the light beam. A pinhole aperture with a 100 μm diameter limits the entrance pupil size. The collimated beam is then focused by MLA_2_ (subunit size: 300 μm, focal length: 5 mm), which is carefully positioned relative to the MLAG to achieve optimal focusing. After passing through the MLAG, light at each wavelength is diffracted and focused at different positions on the image plane.

**Figure 5:**
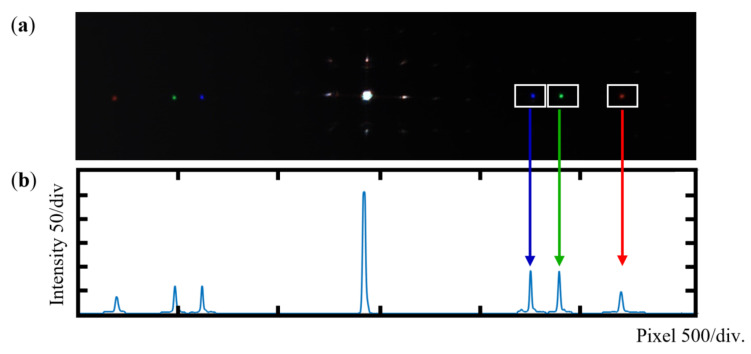
Spectrometer performance test: (**a**) spectrometer imaging results; (**b**) spectral signal extraction.

When the beam is aligned perpendicularly to a specific MLAG microlens subunit, the spectrometer parameters are fixed. The three wavelengths are sequentially focused onto the CMOS detector, where 0th-order and ±1st-order diffraction spots are simultaneously captured. As shown in Figure 5a, only the +1st-order diffraction pattern is displayed due to its symmetry with the −1st order; unless otherwise noted, the +1st-order signal is used for analysis throughout this work. Since raw spectral data may be affected by stray light, a simple filtering step is applied to remove background noise. The intensity distribution is then extracted, and the diffraction spot position is determined using a centroid-based method, as illustrated in Figure 5b.

To evaluate the performance across multiple channels, a 5×5 array of microlens subunits was tested, yielding 25 sets of raw data. Based on the symmetry of ±1st-order diffraction, 23 valid datasets were retained for analysis. The directly computed 3σ positional deviation across the focal spots was found to be in the order of several hundred pixels.

#### 3.3.2. Offset Correction

**Figure 6:**
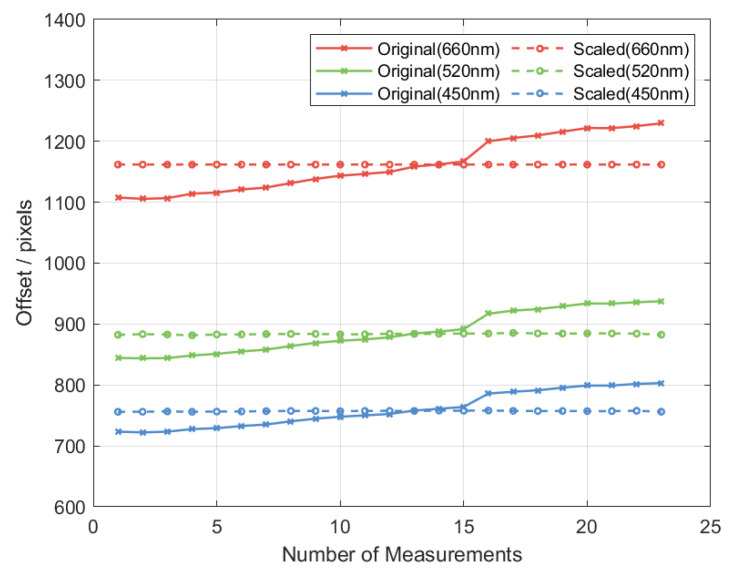
Data correction results.

Due to the movement of the microlens array grating relative to the camera during the experiment, the distance between the imaging plane and the grating also changed, resulting in a variation in the overall image size. The solid lines in Figure 6 present the original measurement data arranged in the order in which the experiments were conducted. It can be observed that, as the experiment progresses, the diffraction shifts corresponding to each wavelength increase simultaneously, indicating that the images captured on the CMOS sensor have undergone a consistent scaling in size.

To compensate for this variation, all images were rescaled. The average displacement of the +1st-order red diffraction spot (660 nm) was selected as a reference, and each image was scaled so that the red +1st-order spot displacement was normalized to 1161.8 pixels. After normalization, the pixel center distances for the 520 nm and 450 nm diffraction spots were calculated. As shown by the dashed lines in Figure 6, this correction effectively reduced deviation across images. The mean pixel center distance for the 520 nm diffraction spot was measured to be 887.0 px, with a repeatability (3σ) of 2.72 px. For the 450 nm diffraction spot, the mean pixel center distance was 759.9 px, with a repeatability (3σ) of 1.91 px.

According to the calibration method described in Section 3.1, the fitted parameters obtained after calibration are $a_{0}=0.000699$, $a_{1}=1.138406$, and $a_{2}=106.074939$. Based on these parameters, the spectral widths corresponding to the pixel positions at different test wavelengths are calculated as follows: 0.49 nm at 660 nm, 0.54 nm at 520 nm, and 0.57 nm at 450 nm. The standard deviations (σ) of the pixel center distances for the 520 nm and 450 nm laser sources are 0.45 nm and 0.38 nm, respectively.

#### 3.3.3. Spot Image Enhancement Processing

**Figure 7:**
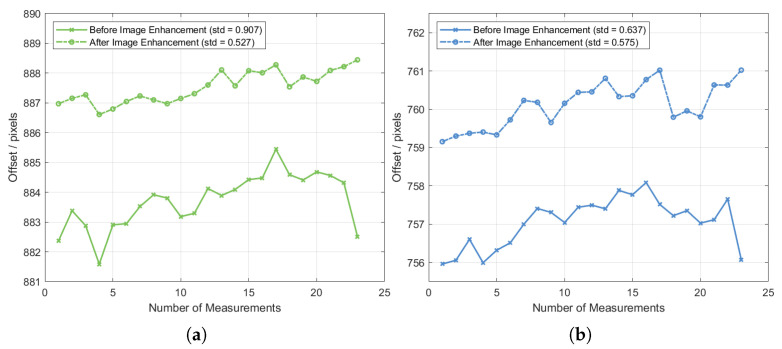
Image enhancement effects: (**a**) 520 nm; (**b**) 450 nm.

In the experiment, the outer regions of the diffraction spots exhibited low brightness and were susceptible to background noise, leading to a low signal-to-noise ratio. Moreover, the spots were not perfectly circular. To improve spot localization accuracy, an image enhancement algorithm was applied to suppress the brightness of the outer regions and emphasize the high-energy spot centers for centroid detection. After enhancement, the effective spot size was reduced, and centroid positions were recalculated and rescaled. The revised pixel center distances for the +1st-order diffraction spots of different wavelengths are shown in Figure 7. The standard deviations of the enhanced 520 nm and 450 nm spots were 0.53 px and 0.58 px, corresponding to spectral widths of 0.26 nm and 0.35 nm, respectively.

It should be noted that this error includes not only the localization uncertainty of the 520 nm and 450 nm spots but also the localization error of the 660 nm reference spot, since all measurements were normalized assuming its position was accurate. In scenarios where the relative position between the MLAG and CMOS sensor is fixed, the achievable precision of the system can be further improved.

**Figure 8:**
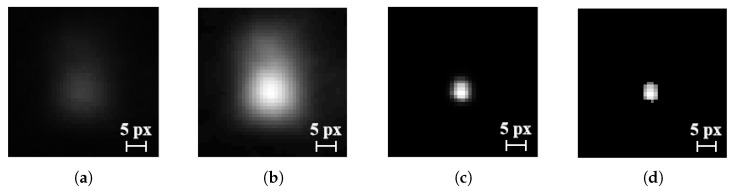
Image enhancement process: (**a**) original grayscale image; (**b**) contrast enhancement; (**c**) nonlinear transformation; and (**d**) filtering out low-brightness pixels.

The image enhancement procedure is as follows:

1. Contrast enhancement for each spot and its local background using linear mapping:
  (5) $$y=255\cdot \frac{x-min}{max-min}$$
  where min and max denote the minimum and maximum brightness in the selected region. The result is shown in Figure 8b.
2. Nonlinear filtering of brightness using:
  (6) $$y=\frac{1}{1+e^{k(x-c)}}$$
  where *k* and *c* are filter parameters. For *k* = 20 and *c* = 0.95, the enhanced image is shown in Figure 8c.
3. Thresholding to remove low-brightness pixels. The filtered result is shown in Figure 8d.

The choice of filtering parameters also impacts the measured standard deviation of diffraction spot positions, as shown in Table 2. Increasing suppression of peripheral brightness reduces the spot width but tends to increase the standard deviation of pixel center distances. This suggests that excessive emphasis on the spot core while ignoring edge brightness reduces centroid stability. Based on the third filtering method, the spot’s full width at half maximum (FWHM) is approximately 5.9 pixels, which corresponds to a single-channel resolution of 3.0 nm at 520 nm wavelength and 3.5 nm at 450 nm wavelength.

To enable multi-channel parallel detection while avoiding overlap and aliasing between diffracted spots from different channels, the most straightforward solution is to use time-division multiplexing. Because a one-dimensional grating produces diffraction in only one direction, channels arranged in columns perpendicular to the diffraction direction can be detected simultaneously without crosstalk. Furthermore, columns separated by specific intervals can also prevent overlapping, allowing multiple channels to be measured in parallel. An even more effective approach is to introduce a small in-plane angle between the microlens array surface and the one-dimensional grating during MLAG fabrication. This adjustment ensures that the direction of diffractive displacement is no longer parallel to the arrangement of microlens array elements, thereby enabling completely non-overlapping, simultaneous multi-channel detection across the entire MLAG.

## 4. Conclusions

To address the need for high-density integration in multi-channel parallel spectroscopic analysis, this work proposes an array-based spectrometer architecture utilizing a microlens array grating (MLAG). By integrating microlenses and a grating onto both sides of a single substrate, simultaneous focusing and dispersion are achieved. The system further optimizes the matching of the incident beam divergence angle using a dual-microlens and aperture assembly, and employs CMOS-based parallel signal acquisition technology. Experimental results indicate that the coefficients of variation for spot spacing in the x and y directions are as low as 1.25% and 1.11%, respectively. The spectral repeatability precision is better than 1.0 nm, with the standard deviation of wavelength shift reduced to as low as 0.26 nm after image enhancement. The spectrometer achieves a resolution of up to 3.0 nm. With the proposed multi-channel parallel detection strategy, the system is theoretically capable of supporting up to 2070 parallel acquisition channels within a 10 mm × 10 mm unit area. This design provides a high-precision, high-throughput solution for array-based MicroLED wafer inspection.

## Figures and Tables

**Table 1:** Uniformity of the MLAG surface (pixels).

No.	X Interval	Y Interval
1	564	563
2	577	574
3	573	576
4	576	568
5	584	580
6	575	573
CV (%)	1.25	1.11

**Table 2:** Comparison of the standard deviation of diffracted light shift under different filtering effects (pixels).

Parameter Combination	Full Width at Half Maximum		SD of Pixel Center Distance	
	Mean	SD	520 nm	450 nm
1	10.565	1.626	0.527	0.575
2	7.957	1.518	0.548	0.594
3	5.913	1.608	0.552	0.585

## Data Availability

The datasets presented in this article are not readily available because the data are part of an ongoing study. Requests to access the datasets should be directed to fy-zhao24@mails.tsinghua.edu.cn.
